# Impact of palmiped farm density on the resilience of the poultry sector to highly pathogenic avian influenza H5N8 in France

**DOI:** 10.1186/s13567-023-01183-9

**Published:** 2023-07-10

**Authors:** Billy Bauzile, Benoit Durand, Sébastien Lambert, Séverine Rautureau, Lisa Fourtune, Claire Guinat, Alessio Andronico, Simon Cauchemez, Mathilde C. Paul, Timothée Vergne

**Affiliations:** 1 IHAP, Université de Toulouse, INRAE, ENVT, Toulouse, France; 2 Laboratory for Animal Health, French Agency for Food, Environmental and Occupational Health and Safety (ANSES), University Paris-Est, 14 rue Pierre et Marie Curie, 94700 Maisons-Alfort, France; 3 Direction Générale de l’Alimentation, Paris, France; 4 Mathematical Modelling of Infectious Diseases Unit, Institut Pasteur, Université de Paris Cité, CNRS UMR2000, 75015 Paris, France

**Keywords:** Bird flu, mechanistic model, simulations, prevention, control, poultry

## Abstract

We analysed the interplay between palmiped farm density and the vulnerability of the poultry production system to highly pathogenic avian influenza (HPAI) H5N8. To do so, we used a spatially-explicit transmission model, which was calibrated to reproduce the observed spatio-temporal distribution of outbreaks in France during the 2016–2017 epidemic of HPAI. Six scenarios were investigated, in which the density of palmiped farms was decreased in the municipalities with the highest palmiped farm density. For each of the six scenarios, we first calculated the spatial distribution of the basic reproduction number (*R*_0_), i.e*.* the expected number of farms a particular farm would be likely to infect, should all other farms be susceptible. We also ran in silico simulations of the adjusted model for each scenario to estimate epidemic sizes and time-varying effective reproduction numbers. We showed that reducing palmiped farm density in the densest municipalities decreased substantially the size of the areas with high *R*_0_ values (> 1.5). In silico simulations suggested that reducing palmiped farm density, even slightly, in the densest municipalities was expected to decrease substantially the number of affected poultry farms and therefore provide benefits to the poultry sector as a whole. However, they also suggest that it would not have been sufficient, even in combination with the intervention measures implemented during the 2016–2017 epidemic, to completely prevent the virus from spreading. Therefore, the effectiveness of alternative structural preventive approaches now needs to be assessed, including flock size reduction and targeted vaccination.

## Introduction

Managing avian influenza epidemics has become a crucial challenge for the long-term sustainability of the European poultry sector. In the last decade, Europe has experienced four major epidemics of highly pathogenic avian influenza (HPAI), resulting in severe socioeconomic consequences in the poultry sector. As a case in point, the 2021–22 episode (subtype H5N1) has been the most devastating yet, with over 2400 reported outbreaks throughout Europe. Over the previous three epidemics, France was one of the most impacted countries in Europe, with approximately 500 reported outbreaks in 2016–17 (H5N8) and in 2020–21 (H5N8) and more than 1300 in 2021–22 [1]. In accordance with European regulations, the French government implemented strict control measures to control virus spread, including the culling of infected flocks, preventive culling of at-risk flocks, movement restrictions in affected zones and pre-movement testing of duck flocks.

Retrospectively, epidemiological studies have demonstrated the pivotal role that palmiped farms have played in France during these epidemics, particularly with the H5N8 subtype. Guinat et al. [2] used a statistical approach to show that the HPAI H5N8 outbreaks (2016–2017) were much more likely to occur in areas with high density of farms raising ducks. This finding was consistent with the conclusions of a mechanistic modelling study [3] that highlighted the importance of local transmission between poultry farms and the higher susceptibility and infectivity of palmiped farms (e.g. ducks and geese) as compared to galliform farms (e.g. chickens, laying hens, quails, etc.) regarding H5N8 clade 2.3.4.4.

Important efforts have been devoted in the subsequent years to improve external and internal biosecurity practices in French poultry farms, with the aim of preventing the risk of HPAI occurrence and its negative impacts [4]. However, the repeated occurrence of HPAI epidemics made a wide range of stakeholders of the poultry sector as well as decision-makers face the vulnerability of the French poultry sector with regards to HPAI. Indeed, they demonstrated cruelly that the major improvements on biosecurity implemented all along the poultry chain had remained insufficient to control recent strains of HPAI viruses.

Livestock farm density is regularly emphasised as a key factor driving highly contagious livestock disease transmission dynamic, as was shown for bluetongue [5], classical swine fever [6] or foot-and-mouth disease [7]. For HPAI, poultry farm density has historically been shown to be associated with the risk of occurrence of HPAI outbreaks [8]. Consequently, HPAI intervention strategies based on the reduction of poultry farm density during HPAI epidemics have been assessed on numerous occasions, as highlighted in a recent systematic review [9]. Indeed, several studies have looked at the effect of artificially reducing poultry farm density as a reactive measure to HPAI spread by implementing preventive culling around infected premises [10–13], sending poultry houses to processing earlier than the normal scheduled date [14] or imposing a ban on restocking on emptied farms [11]. However, all these studies looked at the implementation of these measures during the course of an epidemic in reaction to HPAI spread. To the best of our knowledge, looking at the reduction of poultry farm density as a preventive measure to improve the system resilience against HPAI has not been considered yet.

This study aimed at providing quantitative evidence of the impact a decrease of palmiped farm density in highly dense areas could have on the resilience of the French poultry sector to HPAI outbreaks. To do so, we compared the epidemiological impact of HPAI spread (in terms of epidemic size, basic and effective reproduction numbers) under different scenarios of palmiped farm density reduction.

## Materials and methods

### Underlying mechanistic model

To address this question, we used a farm-based mechanistic spatial model that was previously calibrated to the observed spatio-temporal distribution of HPAI H5N8 outbreaks in France during the 2016–2017 epidemic wave. The model and its calibration are described in detail in [3]. Briefly, the model considered that each farm could pass through four successive infection states, being susceptible, latent (infected but not yet infectious), infectious and recovered (culled). The model assumed that the force of infection ${\lambda }_{i}\left(t\right)$ exerted on a given susceptible farm *i* at time *t* was given by

1 $${\lambda }_{i}\left(t\right)=\sum_{j}{\lambda }_{j\to i}\left(t\right).Ind[j \,infectious \,at \,t]+{{\lambda }_{i}}^{ext}$$

where *Ind* is the indicator function, ${{\lambda }_{i}}^{ext}$ is an external force of infection accounting for infection sources other than the infectious farms located at less than 15 km from farm *i* (e.g. infectious wild birds and farms located further away), and ${\lambda }_{j\to i}\left(t\right)$ is a frequency-dependent force of infection exerted by farm *j* on farm *i* at time *t*, given by

2 $${\lambda }_{j\to i}\left(t\right)={\psi }_{j}.{\varphi }_{i}.{\alpha }_{SZ}\left(i,j,t\right).\frac{\beta (t)}{{{N}_{j}}^{15km}}.Ind\left[{d}_{ij}<15km\right]$$

where ${\psi }_{j}$ is the relative infectivity of farm *j* (with ${\psi }_{j}=1$ for palmiped farms and ${\psi }_{j}=\psi$ for galliform farms), ${\varphi }_{i}$ is the relative susceptibility of farm *i* (with ${\varphi }_{i}=1$ for palmiped farms and ${\varphi }_{i}=\varphi$ for galliform farms), ${\alpha }_{SZ}\left(i,j,t\right)$ is a multiplicative term to account for changes in transmission for farms located in the surveillance zones (SZ) implemented around infected premises (with ${\alpha }_{SZ}\left(i,j,t\right)=1$ if both farms *i* and *j* do not belong to a surveillance zone at time *t* and ${\alpha }_{SZ}\left(i,j,t\right)=\alpha <1$ otherwise), $\beta \left(t\right)$ is a time-space-varying between-farm transmission rate, ${{N}_{j}}^{15km}$ is the number of farms within 15 km from farm *j*, and $Ind[{d}_{ij}<15km]$ being an indicator function taking the value 1 if the Euclidean distance between farms *i* and *j* (${d}_{ij}$) was smaller than 15 km and 0 otherwise. Note that the force of infection did not account explicitly for live-duck movements nor for the transit of vehicles used for these movements, in accordance with previous network analyses that concluded that these transmission routes had generated only very few transmission events during the 2016–2017 epidemic [15, 16]. Also, the transmission rate was considered space–time dependent to allow the Landes department being associated with specific transmission rates, with three different values corresponding to three different time periods (see Table 1), which produced a good fit to the data [3]. Also, in an exploratory work, Andronico et al. [3] investigated density-dependent versions of the model associated with a smoothed distance kernel to penalise the transmission rate as a function of the distance between farms as in [17, 18]. The fit of this alternative model to the data was really poor, justifying the use of a frequency-dependent force of infection associated with a step-function to model local spread. The cut-off distance of 15 km was found to be the one with the strongest support by the data. Andronico et al. [3] further assumed that the external force of infection exerted on galliform and palmiped farms, represented by ${{\lambda }_{i}}^{ext}$ in Equation 1, was defined as ${{\lambda }_{i}}^{ext}={\beta }_{ext}.{\varphi }_{i}$, with ${\varphi }_{i}$ the relative susceptibility of farm *i* (see above) and ${\beta }_{ext}$ being a constant external transmission rate exerted on palmiped farms. Parameters $\psi$, $\varphi$, ${\alpha }_{SZ}$, $\beta \left(t\right)$ and ${\beta }_{ext}$ were estimated by fitting the model to the observed outbreaks during the 2016–2017 epidemic wave and are listed in Table 1. Upon infection, farms were assumed to remain in the latent state for an average duration of one day before moving to the infectious state, in which they stayed for an average duration of seven days before being considered removed permanently through the culling of the flock. Further details on the model and assumptions can be found in the original paper [3].

**Table 1:** **Inferred model parameter values as estimated in Andronico et al**. [3]

Parameter	Definition	Median (95%CI)
$\phi$	Relative susceptibility of galliform farms	0.20 (0.15, 0.27)
$\psi$	Relative infectivity of galliform farms	0.39 (0.10, 0.85)
${\alpha }_{SZ}$	Effect of surveillance zones on transmission rate	0.58 (0.42, 0.80)
${\beta }_{0}$	Daily transmission rate in all departments but Landes	0.23 (0.16, 0.31)
${\beta }_{1}$	Daily transmission rate in Landes before 22 Jan 2017	0.31 (0.20, 0.47)
${\beta }_{2}$	Daily transmission rate in Landes between 22 Jan 2017 and 11 Feb 2017	0.53 (0.37, 0.72)
${\beta }_{3}$	Daily transmission rate in Landes after 11 Feb 2017	0.28 (0.18, 0.40)
${\beta }_{ext}$	External transmission rate (10^–4^)	0.86 (0.62, 1.15)

### Definition of the density scenarios

**Figure 1:**
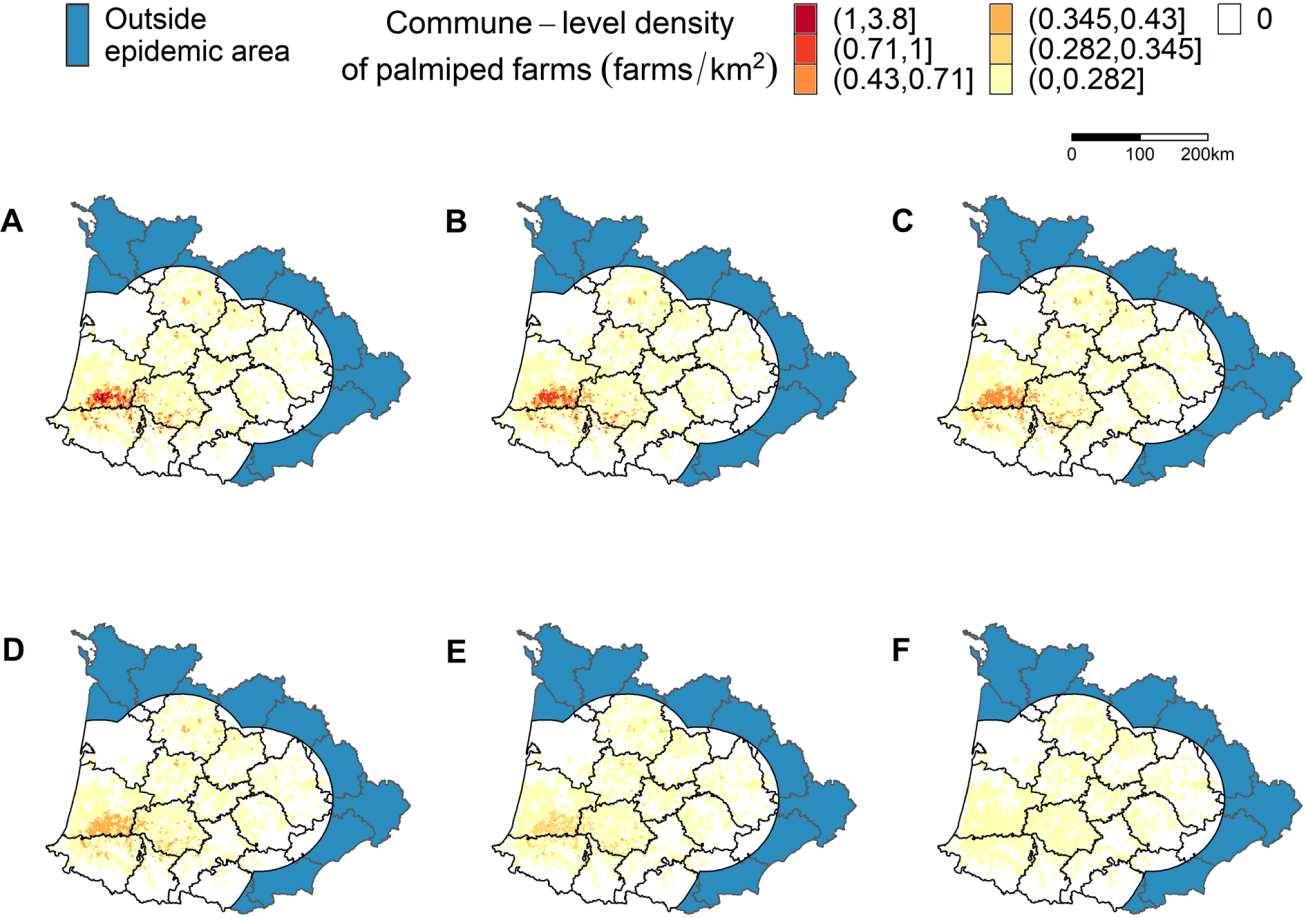
**Distribution of palmiped farm density in the Southwest region of France, for each scenario**. The six scenarios correspond to a simulated reduction of palmiped farm density in the 0% (**A**), 2% (**B**), 5% (**C**), 10% (**D**), 15% (**E**) and 20% (**F**) of the municipalities with the highest palmiped farm density. Note that the category thresholds in the legend correspond to the density thresholds used to define the scenarios. For instance, in scenario B, the densities of the 33 (2%) municipalities with densities between 1 and 3.8 palmiped farms/km^2^ were all reduced to densities of 1 farm/km^2^. Thus, all municipalities in scenario B had densities below 1 farm/km^2^.

We defined the study region as the area located within 100 km from any farm that became infected during the 2016–2017 epidemics (Figure 1). In that region, we further defined six scenarios of palmiped farm density. The baseline scenario considered all 8379 commercial poultry farms, including 4188 galliform and 4191 palmiped farms, as used in [3] to estimate transmission parameters. The five other scenarios were defined based on discussions with the French administration. They simulated a decrease of palmiped farm density in the municipalities (smallest French administrative unit having a median surface of 10 km^2^) with the highest palmiped farm density, as they were shown to be at higher risk of HPAI occurrence [1, 2]. To do so, we selected the 2, 5, 10, 15 and 20% of the municipalities with the highest palmiped farm density. Then, for each scenario, we used the lowest palmiped farm density of these selected municipalities as a threshold to reduce the palmiped farm density in the municipalities with a higher density, by randomly removing palmiped farms until the density reached the threshold (Figure 1). This random removal of farms was repeated for every model run. These scenarios represented respectively a removal of 62, 188, 477, 648 and 825 palmiped farms in 33, 80, 174, 256 and 341 municipalities, which accounted for 0.7%, 2.2%, 5.7%, 7.7% and 9.8% of the total number of poultry farms, respectively.

### Estimation of the basic reproduction number (*R*_0_)

For each of the six scenarios, we first calculated the basic reproduction number (*R*_0_) for each farm, i.e. the expected number of farms a particular farm would be likely to infect, should all other farms be susceptible. For a given farm *j*, *R*_0*j*_ was defined as:

3 $${R0}_{j}=\sum_{i}\left(1-\mathit{exp}\left(-\delta *{\lambda }_{j\to i}\right)\right)$$

where $\delta$ is the duration of the infectious period for a farm (in days) and ${\lambda }_{j\to i}$ is the daily force of infection exerted by an infectious farm *j* on a susceptible farm *i*, as defined in accordance with [3] by:

4 $${\lambda }_{j\to i}\left(t\right)={\psi }_{j}.{\varphi }_{i}.\frac{\beta }{{{N}_{j}}^{15km}}Ind\left[{d}_{ij}<15km\right]$$

where $\beta$ is a constant ${\beta }_{0}$ as defined in Table 1. Note that the term ${\alpha }_{SZ}\left(i,j,t\right)$ is no longer part of the expression of ${\lambda }_{j\to i}(t)$ since the calculation of *R*_0_ assumed that all farms were susceptible and therefore that no surveillance zones were implemented. Also, it is worth noting that *R*_0_ did not integrate the external force of infection, so that, by construction, it only accounted for the infection events resulting from between-farm transmission within 15 km from source farms. For each scenario, the posterior distribution of *R*_0*i*_ was determined by randomly sampling 500 values in the posterior distributions of the parameters, as established in [3] and summarised in Table 1. The spatial distribution of *R*_0_ values was smoothed using ordinary kriging method from gstat package [19] to interpolate the *R*_*0*_ from the point data to the rest of the area and displayed using R software version 4.0.2 [20].

### Estimation of the epidemic trajectories and of the effective reproduction number (*R*_e_)

We then investigated the impact that the reduction in palmiped farm density would have had on the 2016–2017 epidemic dynamics. To do so, we ran 500 stochastic simulations of the model for each scenario, assuming a force of infection as defined in Equation 1 and parameter values drawn from their posterior distributions as summarised in Table 1 [3]. We initialised the model similarly for each simulation with the observed index case being set to the exposed state and with all other flocks being considered susceptible. For a given scenario, palmiped farms to be removed from the population were re-sampled randomly at each model run, as described above. The model accounted for the control strategies implemented during the 2016–2017 epidemic, including culling and removal of infected flocks at the end of the infectious period, implementation of surveillance and protection zones around infected farms (SZ and PZ), enhancement of biosecurity measures in the SZ [accounted for by the parameter ${\alpha }_{SZ}$ in Equation (2)], and preventive culling of palmiped flocks in the PZ and of all poultry flocks within 1 km of infected premises starting in early January, as defined in [3]. Note that the parameter ${{N}_{j}}^{15km}$, representing the number of farms within 15 km of infectious farm *j* in Equation 2, varied across scenarios depending on which palmiped farms were removed, and was updated continuously during a simulation to account for the “removal” of farms due to culling of infected flocks and preventive culling. For each simulation, we reconstructed the transmission tree of the epidemic (i.e. we recorded who infected whom), from which we calculated, for each farm, the number of secondary infections they generated over the course of their infectiousness (i.e. until culling). From this, we calculated, for each day, the mean number of secondary infections generated by farms that became infected on that day and averaged it using a moving seven-day time window. This allowed the estimation of the effective reproduction number (*R*_e_), i.e. the time-varying expected number of farms a particular farm would be likely to infect, while accounting for the implementation of control strategies. Similar to the computation of the *R*_0_, the *R*_e_ did not account to the infection events that were due to the external force of infection. The different scenarios were compared using the 50% and 95% prediction intervals (PI) of the simulated daily incidence and estimated *R*_e_ values. Note that because the external force of infection seeds new infections in the population at a constant rate, using the expected duration of the epidemics was not relevant to compare the scenarios.

## Results

**Figure 2:**
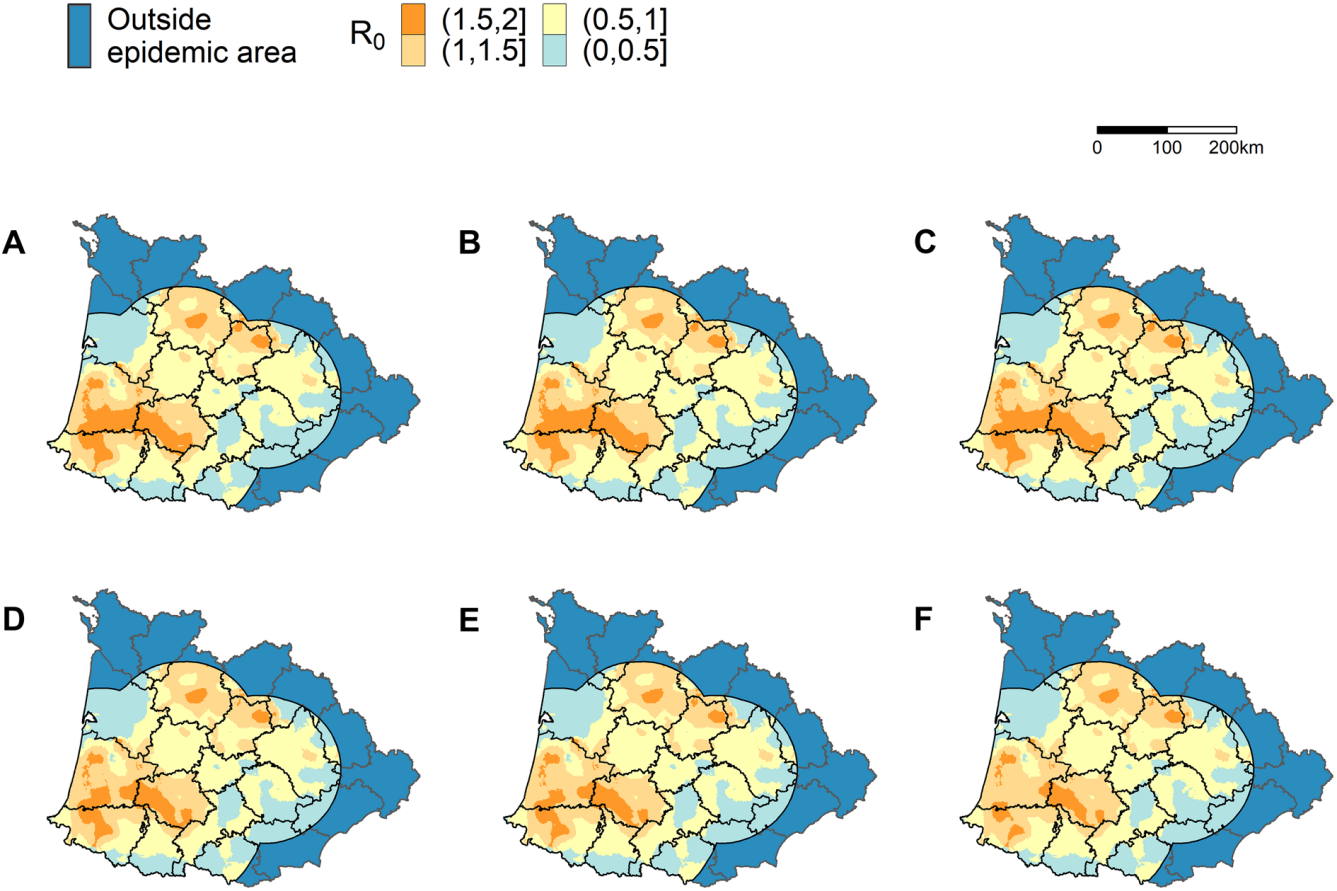
**Smoothed spatial distribution of the basic reproduction number** (R_0_) **for each scenario**. The six scenarios correspond to a simulated reduction of palmiped farm density in the 0% (**A**), 2% (**B**), 5% (**C**), 10% (**D**), 15% (**E**) and 20% (**F**) of the municipalities with the highest palmiped farm density.

The spatial distribution of *R*_0_ in the Southwest region of France for the six different scenarios is represented in Figure 2. Our estimates suggest that reducing the density of palmiped farms in the densest municipalities has an impact on the spatial distribution of *R*_0_: the extent of the geographical areas with high *R*_0_ values (>1.5). However, as shown in dark orange in Figure 2, the size of the area with *R*_0_ > 1.5 starts to decrease in size only from scenario D (reduction of the palmiped farm density in the 10% densest municipalities). It has to be noted that even when the farm density was reduced in the 20% densest municipalities (Figure 2F), i.e*.* when more than 800 palmiped farms were removed from the baseline population, *R*_0_ remained higher than 1.5 in a relatively wide region, including the municipalities where the density was reduced, suggesting that reducing palmiped farm density would not prevent viral spread without the implementation of surveillance and intervention strategies.

**Figure 3:**
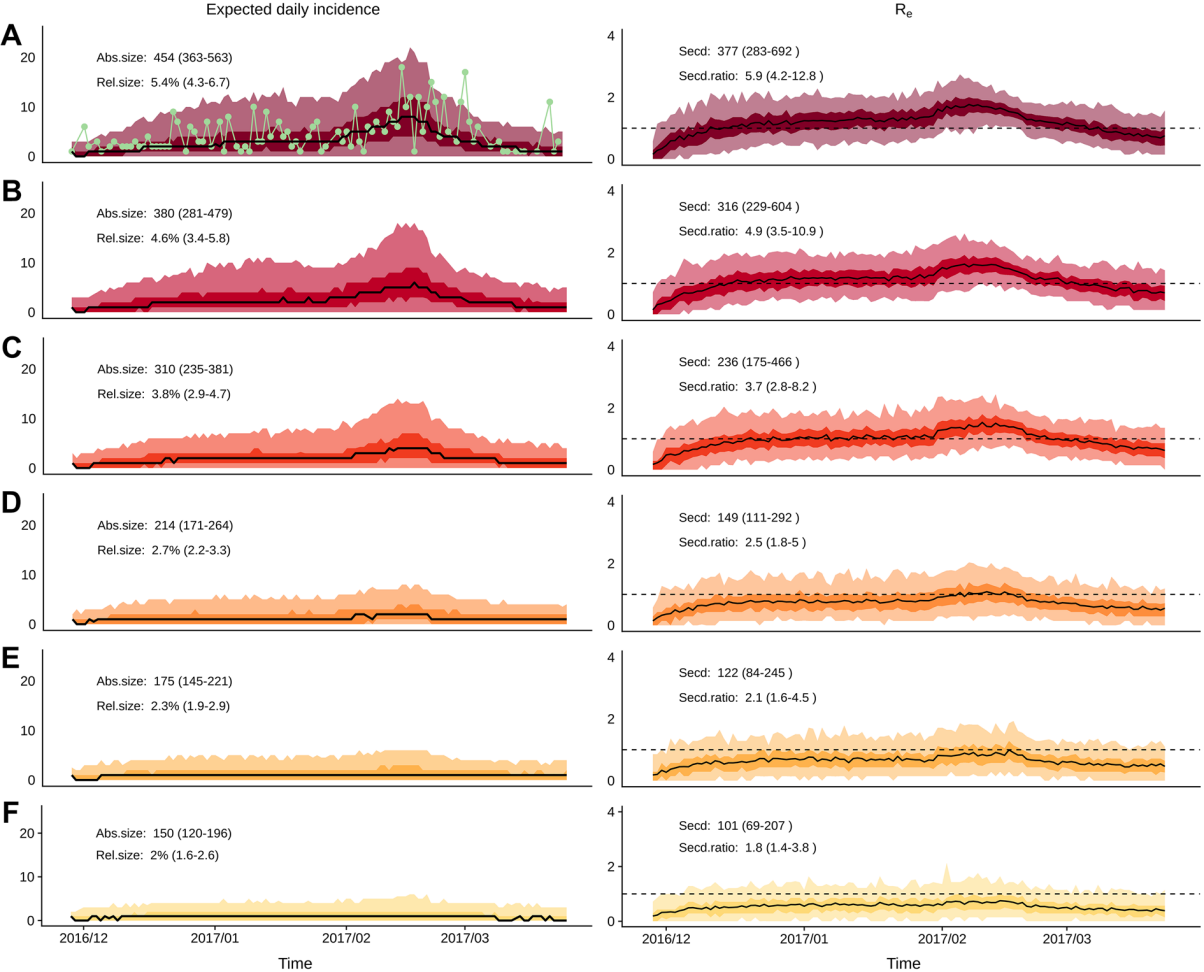
**Expected dynamic of HPAI outbreaks in France during the 2016–2017 epidemic for each scenario**. Left: daily incidence of HPAI outbreaks in France during the 2016–2017 epidemic (left); right: time-varying effective reproduction number (Re, right). The six scenarios correspond to a simulated reduction of palmiped farm density in the 0% (**A**), 2% (**B**), 5% (**C**), 10% (**D**), 15% (**E**) and 20% (**F**) of the municipalities with the highest palmiped farm density. In the top left panel, the green line shows the observed farm-level daily incidence during the 2016–2017 epidemic. In each panel of the left column, solid black lines represent the median daily incidence while the darker and lighter envelopes depict their 50% and 95% prediction intervals, respectively. The numbers inserted in the left plot areas represent the absolute (Abs.size) and relative (Rel.size) expected epidemic sizes and their 50% prediction intervals. In each panel of the right column, solid lines represent the mean Re while the darker and lighter envelopes depict their 50% and 95% prediction intervals, respectively. The numbers inserted in the right plot areas represent the number of farm-to-farm transmission events (Secd) and the average number of farm-to-farm transmission event per farm infected via external sources (Secd.ratio) and their 50% prediction intervals. This figure was based on 500 stochastic simulations from the model for each scenario with the same initial conditions, with parameter values drawn from their posterior distribution (including the external transmission rate assumed to be the same as during the 2016–2017 epidemic) and with the control strategies implemented during the 2016–2017 epidemic.

As illustrated in Figure 3, when accounting for the control strategies implemented during the 2016–2017 epidemic, decreasing palmiped farm density in the densest municipalities would have had a substantial impact on the epidemic. Indeed, it is expected that reducing the palmiped farm density in the 20% densest municipalities would have decreased both the expected final epidemic size—from 454 (95%PI: 363–563) to 150 outbreaks (50%PI: 120–196)—and the expected final proportion of infected farms—from 5.4% (95%PI: 4.3–6.7) to 2.0% (95%PI: 1.6–2.6). However, even in the most drastic scenario, the epidemic would still have led to a total of 150 outbreaks (50%PI: 120–196), which remains substantial. Therefore, given the model assumptions, results suggest that none of the investigated scenarios would have totally curbed the epidemic.

The reconstructed transmission trees of the simulated epidemics revealed that every farm that became infected from an external source of infection, corresponding to between 58 and 66 farms depending on the simulation, led to an average of 5.9 subsequent farm infections (95%PI: 4.2–12.8) in the baseline scenario and that this decreased to 1.8 farm infections (95%PI: 1.4–3.8) in the most stringent scenario. Note that these numbers of secondary infections were calculated for all infection generations that followed the occurrence of an infection from an external source of infection. Figure 3 demonstrates how the temporal evolution of the *R*_e_ values varied between scenarios. In the intermediate scenario (reduction of the palmiped farm density in the 10% densest municipalities through the removal of 477 palmiped farms), each time step had at least 50% of the estimated *R*_e_ below 1, allowing us to conclude with 50% confidence that *R*_e_ would have been below 1 throughout the epidemic (Figure 3). Using a similar reasoning, the most stringent scenario (reduction of the palmiped farm density in the 20% densest municipalities through the removal of 825 palmiped farms) led to a 75% confidence that *R*_e_ would have been below 1 throughout the epidemic (Figure 3). Note that having *R*_e_ below one does not prevent secondary infections from occurring and therefore a substantial number of outbreaks from happening, especially when a substantial number of farms are infected randomly based on the external source of infection, as is the case here. Indeed, assuming a *R*_e_ of 0.8 in an infinite population comprising 100 infected units, one can expect 80 new infections which in turn will generate 64 new infections, which in turn will generate 51 new infections, etc. It is therefore not surprising that in the most stringent scenario, where we are 75% confident that *R*_e_ would have been below 1 throughout the epidemic, the final epidemic size is still around 150 outbreaks.

## Discussion

This modelling study provides quantitative evidence that reducing palmiped farm density in the densest municipalities would reduce substantially the vulnerability of the whole poultry sector to HPAI outbreaks. Indeed, we demonstrated that having fewer palmiped farms in the municipalities with the highest palmiped farm densities would provide benefits for the whole poultry sector by decreasing the overall risk for both galliform and palmiped farms. It is worth noting that these results were obtained for the virus that circulated during the winter 2016–2017 (subtype H5N8), which had the particularity to have impacted more heavily the duck sector [2], due to higher susceptibility and infectivity of palmiped farms as compared to galliform farms [3]. Therefore, reducing palmiped farm density may be expected to be less effective for other HPAI viruses for which the susceptibility and infectivity of galliform farms may be greater than palmiped farms.

It is worth noting that, because the contact rate between farms was assumed to be frequency-dependent, the effect of reducing palmiped farm density was not related to a reduction of the contact rate between farms. Under the frequency-dependent formulation, one would expect that reducing the number of farms would only affect the absolute epidemic size (due to fewer susceptible farms being present) and not the relative epidemic size. Instead, we found an effect on the relative epidemic size (Figure 3). Therefore, our results are essentially due to a second mechanism, which involved the local reduction of the ratio between palmiped and galliform farms. Indeed, since palmiped farms were more susceptible to the virus and more infectious [3], the reduction of palmiped farm density in high density municipalities decreased the force of infection exerted on the remaining susceptible farms.

Due to model construction, the infectious pressure exerted by an infectious farm was limited to farms within a 15 km radius. Long-distance transmission events from infectious farms were not explicitly modelled from farm to farm, but were accounted for implicitly by the external force of infection that was considered constant across space and time. Consequently, the largest benefit of reducing palmiped farm density is expected to be for the remaining susceptible farms located in the high density municipalities. The density reduction is not expected to have a direct effect on the risk to farms located in municipalities with lower palmiped farm densities. However, decreasing the overall epidemic size would indirectly reduce the risk of spread towards areas with lower palmiped farm densities.

Although it decreased the reproduction numbers and the epidemic size, we showed that decreasing palmiped farm density, even in combination with the intervention strategies that were implemented in 2016–2017, would not have been sufficient to reduce the transmission rate to levels that would completely prevent the virus from spreading (Figures 2 and 3). However, it is likely that our approach underestimated this density effect for two reasons. First, it was assumed that the timeliness of the intervention strategies that were implemented following the detection of outbreaks (represented in the model by the delay between the onset of infectiousness and the culling of the flock) was constant across scenarios. However, it is likely that reducing the outbreak incidence would limit the risk that the veterinary services become overwhelmed by the number of farms to depopulate or to control, as was shown to be the case for foot-and-mouth disease in various settings [21–23]. In turn, this could improve the timeliness of their intervention and their ability to communicate to farmers, thus reducing the delay before culling. Therefore, we would expect even lower daily incidence, number of farm-to-farm transmission events and Re values (Figure 3) for scenarios B to F. Second, the external forces of infection for palmiped or galliform farms (${{\lambda }_{i}}^{ext}$) were assumed to be constant across the epidemic and estimated based on the epidemiological context of the 2016–2017 epidemic. These two parameters, already included in the original model, were key to represent the long-distance transmission processes that were not captured explicitly by the local farm-to-farm force of infection [3]. In the scenarios B to F, we used the same external forces of infection as in the baseline scenario, although it is likely that the external forces of infection would be positively correlated with the number of active outbreaks and therefore decrease with decreasing number of outbreaks incurred by the reduced palmiped farm density. Consequently, we may have overestimated the expected daily incidence (Figure 3) for scenarios B to F, and thus, the final epidemic sizes. It is worth noting that the number of farm-to-farm transmission events and the *R*_*e*_ values are not likely affected by this assumption, since the transmission trees used to calculate these epidemic statistics only focused on farm-to-farm transmission.

In the real world, implementing a reduction of palmiped farm density in the densest municipalities might not be easy. On the short term, this effect could be achieved by extending the delay between production cycles (from the fattening or slaughtering of the previous batch of ducks to the installation of the following one) which is usually of around three weeks. This would effectively reduce the number of palmiped farms that are active at a given time point. Such strategy could be applied during the high-risk period of HPAI introduction and spread (i.e. autumn and winter), providing palmiped breeders receive a financial compensation by the state to account for their production losses.

Given the reduction of palmiped farm density might not be sufficient and its implementation be likely to raise important socio-economic issues, to further improve the resilience of the poultry sector to HPAI epidemics, it is now paramount to further investigate the effect of other strategies on the virus transmission dynamics. Meadows et al. [7] disentangled the relative impact of farm and livestock density on foot-and-mouth disease epidemic size. They showed that increasing livestock density, i.e*.* increasing the number of cattle per geographical unit, was associated with larger epidemics, and that the effect of farm density on epidemic size increased with livestock density. Therefore, one important question that should now be addressed is related to the impact of palmiped flock size on virus transmission risk. Additionally, during their first production stage, ducks raised for foie-gras have access to large outdoor fields, where they can have direct and indirect contacts with commensal wild birds [24] as well as with neighbouring farms via the environment, potentially contributing to the diffusion of HPAI viruses. However, the impact of outdoor grazing on the virus transmission dynamics still has not been clarified, mainly due to the unavailability of relevant data at the time the model of the 2016–2017 epidemic was developed. Following the 2020–2021 epidemic, they are now available and could be used to reconstruct the 2020–2021 epidemic and assess the impact of these two additional drivers. To do so, the mathematical model that was used in this study should be extended to account for this additional data complexity and adjusted to the most recent HPAI epidemics that occurred in France. Finally, given that the idea of vaccinating poultry against highly pathogenic avian influenza viruses, a long-considered tabooed strategy in Europe, is now being given full consideration [25], it becomes essential to develop modelling approaches that can contribute to defining appropriate vaccination strategies of poultry.

## Data Availability

The code used in this study is publicly available on BB’s GitHub repository: https://github.com/bbauzile/DensityAnalysis_AI.
